# Unusual Presentation of Thrombotic Thrombocytopenic Purpura With Non-ST-Elevation Myocardial Infarction

**DOI:** 10.7759/cureus.29499

**Published:** 2022-09-23

**Authors:** Zaid Gheith, Areej Kilani, Omar Al-Taweel

**Affiliations:** 1 Cardiology, The University of Texas Health Science Center at San Antonio, San Antonio, USA; 2 Internal Medicine, University of Jordan, Amman, JOR; 3 Cardiology, University of Nevada-Las Vegas, Kirk Kerkorian School of Medicine, Las Vegas, USA

**Keywords:** chest pain, thrombocytopenia, ttp, non-st segment elevation myocardial infarction (nstemi), nstemi

## Abstract

Thrombotic thrombocytopenic purpura (TTP) is a multisystem disease characterized by disseminated thrombus formation in the arterioles and capillaries. Patients usually present with weakness, subtle mental changes, fever, and acute kidney injury. Cardiac symptoms, such as chest pain or arrhythmia, have been reported but were rarely the sole presenting symptom. We report the case of a 68-year-old woman with acute non-ST-elevation myocardial infarction who was found to have TTP. Prompt diagnosis of TTP is essential because traditional approaches to manage an acute coronary event, inclusive of dual antiplatelet therapy and percutaneous coronary intervention, might be contraindicated due to an increased risk of bleeding. Early administration of steroids and urgent initiation of plasmapheresis to improve platelet count would be crucial initial steps in the management of these patients.

## Introduction

Thrombotic thrombocytopenic purpura (TTP), a subset of thrombotic microangiopathies, is a group of heterogeneous disorders characterized by disseminated thrombus formation in the arterioles and capillaries resulting in thrombocytopenia, microangiopathic hemolytic anemia (MAHA), and potential end-organ injury [1]. Patients usually present with weakness, pallor, headache, or other subtle mental changes. The usual pentad consisting of fever, thrombocytopenia, MAHA, elevated creatinine, and neurologic symptoms is not always seen [2]. This report describes a patient with TTP who presented with acute non-ST-elevation myocardial infarction (NSTEMI).

## Case presentation

A 68-year-old woman with a past medical history of hypertension, diabetes mellitus, cerebrovascular accident, and peripheral artery disease presented to the emergency department with complaints of intermittent exertional chest pain for two weeks that progressed to occur with minimal activity two days prior to arrival. It was nonradiating and associated with shortness of breath. On physical examination, vital signs were stable. The patient was confused, and her motor strength was 3/5 in the left upper and lower extremities. Examination of other systems was unremarkable.

**Figure 1:**
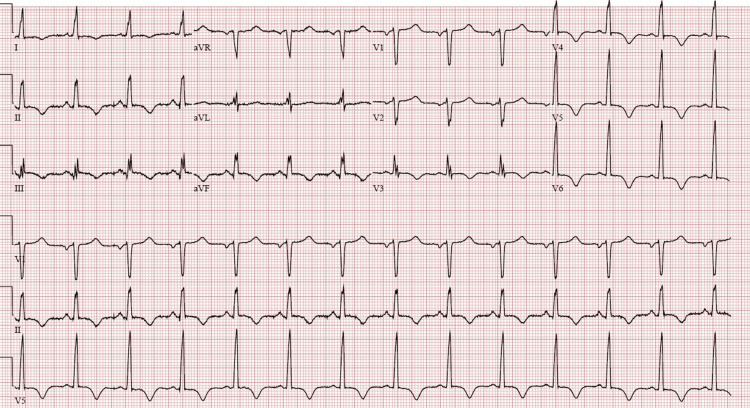
ECG showing T-wave inversion in inferior and lateral leads.

Laboratory investigation revealed anemia, leukocytosis, and thrombocytopenia. Additionally, the patient had high creatinine and evidence of myocardial injury with high troponin I level. Serum lipase was elevated, haptoglobin was decreased, and lactate dehydrogenase level was high. The peripheral blood smear showed schistocytes (four to six per high-power field). A disintegrin and metalloproteinase with a thrombospondin type 1 motif, member 13 (ADAMTS13) activity was low. ADAMTS inhibitor assay was positive pointing toward a high probability of TTP (Table 1). Twelve-lead electrocardiogram showed sinus rhythm with T-wave inversion in inferior and lateral leads but no ST-segment elevation (Figure 1); no prior electrocardiograms were available for comparison. A transthoracic echocardiogram performed on the first day of hospitalization showed moderate-to-severe global left ventricular systolic dysfunction (left ventricular ejection fraction of 30%) with severe hypokinesis of basal-to-mid anteroseptal, basal-to-mid inferoseptal, and basal-to-mid inferior left ventricular wall segments. Computed tomography of the head showed no evidence of brain hemorrhage or infarctions.

**Table 1:** Laboratory investigation WBC, white blood cell; LDH, lactate dehydrogenase; ADAMTS13, A disintegrin and metalloproteinase with a thrombospondin type 1 motif, member 13; N/A, not available.

	Reference range	Admission	Day 3	Day 7
Hemoglobin	12.2-16 g/dL	7.4 g/dL	8.5 g/dL	9.1 g/dL
WBC count	4.0-10.5 × 10^3^/uL	11 × 10^3^/uL	9 × 10^3^/uL	8.7 × 10^3^/uL
Platelet count	140-400 × 10^3^/uL	30 × 10^3^/uL	110 × 10^3^/uL	210 × 10^3^/uL
Creatinine	0.5-1.1 mg/dL	2.4 mg/dL	2.2 mg/dL	0.9 mg/dL
Troponin I	<0.03 ng/mL	35.6 ng/mL	N/A	N/A
Lipase	<150 U/L	1348 U/L	N/A	N/A
Haptoglobin	40-180 mg/dL	5 mg/dL	N/A	N/A
LDH	126-240 IU/L	530 IU/L	N/A	N/A
ADAMTS13	>55%	11%	N/A	N/A

**Figure 2:**
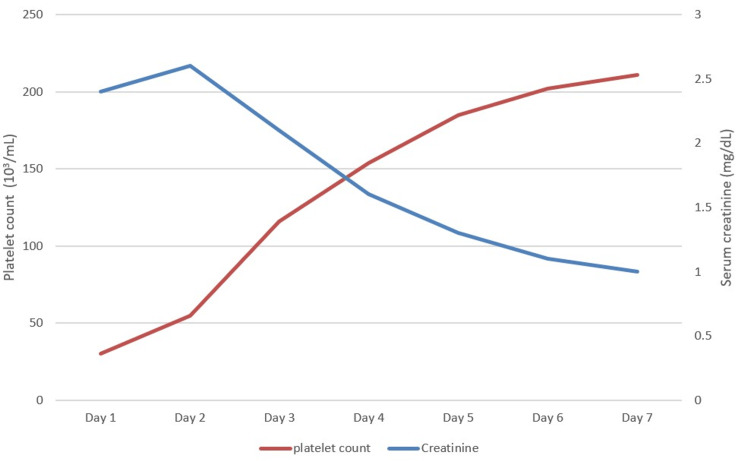
Creatinine and platelet count trend during hospitalization.

Urgent plasmapheresis was performed, and intravenous methylprednisolone was administered on the day of admission while rituximab was started on the following day. Coronary angiography performed on the fourth day of hospitalization showed a right dominant system with 100% occlusion of the proximal posterolateral branch and 80% stenosis of the second obtuse marginal branch but no significant obstructive lesions in the left main, left anterior descending, left circumflex, or right coronary arteries. Conservative medical management with aspirin, clopidogrel, metoprolol, and atorvastatin was initiated. Over a seven-day period since the day of hospital admission, acute kidney injury resolved, and thrombocytopenia improved (Figure 2). The patient remained in the hospital for two weeks, received rituximab and steroids for six weeks, and cyclophosphamide was started thereafter. Plasmapheresis was instituted three times weekly for a total of 12 weeks. Altered mental status resolved and the patient showed significant improvement in her motor strength as well.

## Discussion

Our case report describes a patient who presented to the hospital with an acute coronary syndrome characterized by worsening angina, ischemic changes on electrocardiogram, and elevation of serum troponin level, all consistent with acute NSTEMI. Profound thrombocytopenia on initial laboratory work-up prompted evaluation of the peripheral smear that revealed fragmented red blood cells (schistocytes), which lead to the expedited diagnosis of TTP. It is hard to link a strong causal relation that TTP led to NSTEMI but it would be a challenging situation when both are correlated in the same patient. 

Cardiac involvement in TTP is well known; however, chest pain as the presenting symptom with evidence of NSTEMI is rarely seen. In a systematic review of 13 case series comprising more than 50 patients, none mentioned cardiac symptoms or had clinical cardiac abnormalities [3]. Cardiac manifestations of TTP can range from asymptomatic elevation of cardiac biomarkers to myocardial infarction (MI), arrhythmias, heart failure, myocarditis, or sudden cardiac death. The acute coronary syndrome can occur due to microthrombi formation in small vessels supplying the myocardium, which makes it exceedingly difficult to distinguish from atherosclerotic acute coronary syndrome [4-6].

The exact incidence of cardiac involvement in TTP is unknown [3]. A review of 56 autopsies of patients with TTP revealed extensive cardiac involvement and the presence of platelet-rich thrombi in cardiac vessels despite the rarity of cardiac symptoms in that subset of patients [7].

Several studies have examined the incidence of MI in TTP. In a study of 32 patients with evidence of TTP, 14 patients had evidence of MI. Coronary angiography was performed in only three of these patients and only one patient had significant coronary occlusion [8]. The patient presented in our case report underwent a coronary angiogram that showed significant occlusion in two branch vessels that was not amenable to invasive therapy.

Mortality and disease refractoriness to treatment are considered higher in patients with TTP who have positive cardiac biomarkers. Elevated serum troponin (greater than 0.25 µg/mL) is associated with about a three-fold increase in the risk of death or refractoriness to treatment [9]. The extent of coronary involvement is not well established because not all the patients studied underwent invasive coronary evaluation.

Treatment of MI in the setting of TTP can be challenging. Thrombocytopenia found in these patients can add more complexity to clinical management because initiation of dual anti-platelet therapy (aspirin and clopidogrel or ticagrelor) in thrombocytopenic patients will increase the risk of bleeding. Further, the addition of aspirin to TTP patients has been shown to inhibit platelet aggregation and reduce mortality [10]; however, the role of adding clopidogrel to the treatment regimen is debatable because clopidogrel itself can cause TTP in exceedingly rare cases [11].

Delay in treatment of TTP can lead to rapid progression of the disease and the patient can deteriorate into stupor, coma, cardiac arrest, and death. Early plasma infusion and plasma exchange has reduced the case fatality of TTP from more than 90% to around 10-20% [9]. In the event of acute MI, early heart catheterization and percutaneous coronary intervention are usually approached cautiously as the patient often will have acute kidney injury and severe thrombocytopenia. Invasive coronary intervention can be considered after a few sessions of plasmapheresis allowing for improvement in platelet counts and kidney function [12].

The patient in our study underwent plasmapheresis and received intravenous methylprednisolone urgently. Platelet counts and kidney function started to improve after three sessions of plasmapheresis. Aspirin was then added, and left heart catheterization was safely performed. The coronary angiogram revealed obstructive coronary artery disease in two small coronary branches that were not amenable to intervention.

## Conclusions

Acute coronary syndrome in the presence of TTP is rare but its timely recognition is important for practicing providers because traditional management approaches with dual antiplatelet agents may not be applicable and urgent coronary intervention might not be feasible in this setting. Although the addition of aspirin early in the course of treatment has shown mortality benefits, the need to add a second anti-platelet agent for optimal management of an acute coronary event may worsen the outcome as it could subject the patient to a higher risk of bleeding. Thus, the mainstays of treatment for TTP, inclusive of early steroid administration and therapeutic plasma exchange, should be promptly instituted. Invasive coronary evaluation can be offered after platelet counts and kidney function improve.
